# Exploring the role of information and communication technologies in allergic rhinitis in specialist centers: Patient perspectives on usefulness, value, and impact on healthcare

**DOI:** 10.1002/clt2.12325

**Published:** 2024-01-19

**Authors:** Ivan Cherrez‐Ojeda, Jean Bousquet, Zouina Sarfraz, Azza Sarfraz, Monica Rodriguez Gonzales, Anna Bedbrook, Nelson Rosario, Benjamin Zepeda‐Ortega, Guillermo Guidos, Ulbio Alcivar Molina, Miguel Felix, Emanuel Vanegas, Karla Robles‐Velasco, Luc J. Zimmermann, Antonio W. D. Gavilanes

**Affiliations:** ^1^ Universidad Espíritu Santo Samborondón Ecuador; ^2^ Respiralab Research Group Guayaquil Ecuador; ^3^ Institute of Allergology Charité – Universitätsmedizin Berlin Corporate Member of Freie Universität Berlin and Humboldt‐Universität zu Berlin Berlin Germany; ^4^ Fraunhofer Institute for Translational Medicine and Pharmacology ITMP Allergology and Immunology Berlin Germany; ^5^ University Hospital of Montpellier Montpellier France; ^6^ Research and Publications Fatima Jinnah Medical University Lahore Pakistan; ^7^ Pediatrics and Child Health The Aga Khan University Karachi Pakistan; ^8^ Urticaria Center of Reference and Excellence (UCARE) Department of Allergy Hospital Español de México Mexico City Mexico; ^9^ ARIA & MASK‐air Montpellier France; ^10^ Hospital de Clinicas University of Parana Parana Brazil; ^11^ Pediatric Allergist Private Practice Angeles Lomas Hospital Huixquilucan Mexican State Mexico City Mexico; ^12^ Department of Immunology School of Medicine Instituto Politecnico Nacional Gustavo A. Madero CDMX Mexico City Mexico; ^13^ Hospital Luis Vernaza Servicio de Otorrinolaringología Guayaquil Ecuador; ^14^ School for Oncology & Developmental Biology (GROW) University of Maastricht Maastricht Netherlands; ^15^ Instituto de Investigación E Innovación en Salud Integral Laboratorio de Biomedicina Facultad de Ciencias Médicas Universidad Católica de Santiago de Guayaquil Guayaquil Ecuador

**Keywords:** allergic rhinitis, information and communication technologies, Ecuador

## Abstract

**Introduction:**

Information and communication technologies (ICTs) improve patient‐centered care and are routinely used in Allergic Rhinitis (AR), but patients' preferences and attitudes are unexplored. This study examines AR‐related information preferences and ICT use by AR patients.

**Methods:**

A survey‐based cross‐sectional study was carried out in Ecuador from July to September 2019 in seven centers of reference for allergic disease. Participants were 18 years or older, diagnosed with AR and had access to ICT and the Internet. Descriptive and binomial logistic regressions were performed. A value of less than 0.05 was considered statistically significant.

**Results:**

217 patients were included. 47% (*n* = 102) used ICTs to learn about AR, of which 38.2% (*n* = 83) found it useful. Most of participants (75%, *n* = 164) did not think that ICTs reduce their need to see a doctor. Individuals with poorer quality of life were more likely to utilize ICTs to contact their doctor (OR 1.27, 95% CI 1.04–1.55), and more likely to be interested in AR‐related content (OR 1.23, 95% CI 1.00–1.52). Patients with long‐term AR or other allergies were less likely to use ICTs (OR 0.92 and OR 0.40 respectively). Higher education and lower quality of life may increase AR apps adoption (OR 4.82, 95% CI 1.11–21.00). Academic preparation five‐fold increased ICT use for health provider communication (OR 5.29, 95% CI 1.18–23.72). Mild‐persistent AR enhanced the probabilities of using ICTs to share experiences and communicate with other patients (OR 12.59, 95% CI 1.32–120.35).

**Conclusions:**

Our study emphasizes the importance of tailoring digital resources to patient needs by considering factors such as quality of life, education, and specific subgroups within the AR patient population. Additionally, the findings suggest that while ICTs can play a valuable role in patient education and support, they should complement, rather than replace, traditional medical care for many AR patients.

## 1 INTRODUCTION

Allergic Rhinitis (AR) is a prevalent condition affecting one billion people worldwide and significantly impacting their quality of life.^1^ In Latin American populations, AR is associated with fatigue, irritability, misery, depression, anxiety, impaired cognitive functions, embarrassment,^2^, ^3^ and high direct and indirect costs related to AR^4^ with high rates of work presenteeism and absenteeism reported due to decreased productivity.^4^, ^5^, ^6^ The use of information and communication technologies (ICTs) in healthcare is rapidly increasing, and they offer potential benefits in AR management, including improved patient‐centered care, quality, and education.^7^

Despite the increasing use of ICTs and digitally enabled, patient‐centered care pathways available^8^ for AR monitoring and management,^9^ little is known about patients' preferences and attitudes toward the use of these technologies. Understanding patients' preferences and attitudes towards ICTs in AR management is crucial for the development of effective and acceptable digital tools. It could also help identify potential barriers to technology adoption and inform strategies to overcome them. Therefore, this study aimed to assess patients' perspectives toward AR‐related information as well as the usefulness of ICTs in managing AR in specialized allergy centers, targeting patients with AR.

## 2 MATERIALS AND METHODS

### 2.1 Study design

An anonymous survey‐based cross‐sectional study enrolling 217 patients diagnosed with AR was conducted in accordance with the AR and its Impact on Asthma (ARIA) guidelines. A 22‐item survey was utilized to ascertain the interest in the use of ICT for health‐related purposes.

### 2.2 Setting

This study was carried out in Ecuador from July to September 2019 in seven centers of reference of allergic diseases. We sent the designed questionnaire to the heads of these research centers. The heads then asked their specialists to apply the questionnaire to their patients according to the inclusion criteria described below.

### 2.3 Participants

The recruitment of participants was conducted using a non‐probabilistic sampling method at each site. Every participant was required to satisfy the following inclusion criteria: (i) aged at least 18 years, (ii) with a confirmed diagnosis of AR through skin prick testing by a specialist physician, and (iii) with access to both ICT and the Internet. Patients were excluded if (i) they were without ICT access and active Internet connection, and/or (ii) they had a psychiatric disease, language impairment, or intellectual disability, or (iii) they found it difficult to visualize the questionnaire. Although the sampling technique employed in this study may not encompass the entirety of medical professionals or experts in broader geographical regions, it is a targeted sample approach that gathered data from specialized facilities in Ecuador. These centers primarily focus on the treatment of common allergies and associated disorders, such as AR.

### 2.4 Ethical Considerations

The Institutional Review Board approval (IRB) number is #19–0054 and the method for obtaining informed consent was online. This method for obtaining informed consent was approved by the IRB overseeing the study. All participants provided online informed consent prior to completing the survey.

### 2.5 Outcomes

A 22‐item questionnaire was designed and reviewed by an expert panel of physicians from the research network of AR and Its Impact on Asthma (ARIA). The questionnaire comprised three sections. The first (items 1–13) collected demographic data (age, gender, educational level, area, cellphone ownership, Internet access) and clinical data (time since initial diagnosis of AR, disease severity, associated allergic comorbidities). Educational level was defined as tertiary if the participant had an undergraduate and graduate degree and quaternary if the individual had any postgraduate degree. Regarding disease severity, it was classified/graded based on the latest ARIA guideline recommendations^10^ and divided into “mild” and “moderate‐severe”. The second section (items 14–15), whose answers were applied as an inclusion criterion, assessed access to ICT and an Internet connection.

The third section (items 16–22) assessed the agreement/interests regarding specific statements on the use of ICT. Items 16,17 and 19 were designed to assess in a dichotomous manner (“*yes*” or “*no*”) the agreement regarding the statements: (i) use of ICTs to obtain information related to AR, (ii) usefulness of the information obtained, and (iii) usefulness of ICTs to reduce office consultations. Meanwhile, items 18, and 20–22 were designed to evaluate the level of interest using a 5‐point Likert scale (‘*not interested at all*’, ‘*not very interested*’, ‘*neutral*’, ‘*somewhat interested*’, ‘*very interested*’) regarding the following topics: (i) interest in communicating with their healthcare providers using ICTs, (ii) interest in the content developed and curated by physicians regarding allergy and AR, accessible through ICTs, (iii) interest in the development of a mobile app for the management of AR, and (iv) interest in participating in support groups and reaching other patients with AR to share experiences.

Finally, to assess the quality of life, patients completed the Rhino conjunctivitis Quality of Life Questionnaire (RQLQ). This instrument consists of 28 questions in 7 dimensions (activities, sleep, non‐nose/eye symptoms, practical problems, nasal symptoms, eye symptoms, and emotions), with each question measured on a 7‐point scale (0 = not impaired at all 6 = severely impaired). The overall RQLQ score is the mean of all 28 responses, and the individual domain scores are the means of the items in those domains.

### 2.6 Sample size

The sample size was not calculated as this was an exploratory study.

### 2.7 Statistical analysis

Descriptive statistics were applied for demographic and clinical variables. Continuous data were presented as means and standard deviations if normally distributed, while frequencies were represented as percentages. For both nominal and ordinal data regarding agreement and interests in ICT use for AR purposes, a chi‐square goodness of fit test was applied to determine whether the observed frequencies differed significantly from the expected ones.

Binomial logistic regression was performed to predict the likelihood of participants answering “yes” to questions 16, 17, and 19 based on age, gender, education level, living area, AR type, years with AR, additional allergic conditions, and RQLQ score. Another cumulative odd ordinal logistic regression was performed to determine the effect of the same independent variables on the perception of being interested in ICT use stated in questions 18, 20–22. All these analyses are presented as supplemental files (Table S1 to S6). The data were analyzed using SPSS version 24.0 software, and a significance level of less than 0.05 was used for all tests.

## 3 RESULTS

### 3.1 Demographic characteristics

The study included 250 patients from Ecuador, with a mean age of 30.3 (SD, 13.1) and a female‐to‐male ratio of 2.6:1. Over half of the participants (52.1%, *n* = 113) had achieved tertiary or quaternary education, and almost all (96.8%, *n* = 210) lived in urban areas. Clinical variables showed that the majority (91.7%, *n* = 199) had moderate to severe AR, and the mean RQLQ score was 2.8 (SD, 1.4), with “practical problems” being the most affected domain (X̅ = 3.5; SD, 2.0) (Table 1). Of the 250 participants, 97.6% (*n* = 244) owned electronic devices with ICT access, and 86.8% (*n* = 217) had an active internet connection. The final sample for analysis comprised those with access to both electronic devices and an internet connection (86.8%, *n* = 217).

**TABLE 1:** Demographic and clinical information of the surveyed population (*n* = 217).

Characteristics	Value % (*n*)
Age (mean, SD)	30.3 (13.1)
Gender	
Male	27.6 (60)
Female	72.4 (157)
Education level	
Primary education	3.2 (7)
Secondary education	44.7 (97)
Tertiary/Quaternary education	52.1 (113)
Living area	
Urban	96.8 (210)
Rural	3.2 (7)
Allergic rhinitis severity and pattern	
Mild intermittent	6.5 (14)
Mild persistent	1.8 (4)
Moderate‐severe intermittent	51.6 (112)
Moderate‐severe persistent	40.1 (87)
Years since diagnosis (mean, SD)	5.4 (7.9)
Allergic comorbidity	26.3 (57)
Asthma	8.3 (18)
Atopic dermatitis	8.3 (18)
Allergic conjunctivitis	0.9 (2)
Other	10.6 (23)
RQLQ score (mean, SD)	2.8 (1.4)
Activities (mean, SD)	3.1 (1.6)
Sleep problems (mean, SD)	2.4 (2.1)
Nose symptoms (mean, SD)	3.3 (1.7)
Eye symptoms (mean, SD)	2.2 (1.7)
Non‐nose/eye symptoms (mean, SD)	2.8 (1.7)
Practical problems (mean, SD)	3.5 (2.0)
Emotional problems (mean, SD)	3.0 (2.0)

*Note*: Tertiary education was defined as undergraduate and graduate degree; Quaternary education was defined as any postgraduate degree.

Abbreviation: RQLQ, Rhinoconjunctivitis Quality of Life Questionnaire.

### 3.2 Main data

Nearly half of the participants (47%) reported using ICTs to gather information about AR, with 38.2% finding it useful. Although half of the sample expressed interest in asking medical providers questions about AR through ICTs, most (75.6%) did not believe that these technologies could reduce their need to see a doctor. Interestingly, a significant number of participants showed interest in developing Internet content (60.8%) and an AR‐related app (44.7%) accessible through ICTs. Roughly half of the participants also expressed interest in sharing their experiences related to AR and communicating with other patients with the same condition through ICTs (54.4%).

### 3.3 Binomial and ordinal regressions

Having an additional allergic condition was associated with reduced use of ICTs to obtain information about AR. Of those (47%, *n* = 102) who sought such information, older individuals were more likely to find it useful (OR, 1.16), while those with longer disease histories were less likely to do so (OR, 0.92) (0.84–0.99) (Table 2).

**TABLE 2:** Characteristics of participants who have access to ICTs answering “*Yes*” to queries or expressing interest in ICT use for health‐related purposes in allergic rhinitis.

Variable	Answering “*Yes*” to query or expressing interest in ICT use OR (95% CI)
Have you used ICTs to obtain information about allergic rhinitis? (*n* = 217)	
Additional allergic condition^a^	
Yes	0.40 (0.21–0.78)
Do you think that allergic rhinitis information obtained through ICTs is useful? (*n* = 102)	
Age	1.16 (1.04–1.28)
Years with allergic rhinitis	0.92 (0.84–0.99)
Additional allergic condition	
Yes	11.86 (1.11–127.06)
How interested are you in asking questions to physicians/medical health providers about allergic rhinitis through ICTs? (*n* = 217)	
Mean RQLQ score	1.27 (1.04–1.55)
Education level^b^	
Undergraduate/Graduate—Postgraduate	5.29 (1.18–23.72)
Do you consider that ICTs could reduce the need to see a doctor? (*n* = 217)	
Education level^b^	
Secondary education	0.13 (0.02–0.84)
Undergraduate/Graduate—Postgraduate	0.10 (0.02–0.61)
How interested are you in the development of content (articles, videos, tutorials, etc.) related to allergic rhinitis and allergies y general, created by general practitioners and specialists, accessible through ICTs? (*n* = 217)	
Mean RQLQ score	1.23 (1.00–1.52)
How interested are you in the development of an app designed to manage allergic rhinitis which allows to record your symptoms daily, including drug prescription reminders and general advice about the disease? (*n* = 217)	
Age	0.97 (0.95–0.99)
Mean RQLQ score	1.23 (1.01–1.50)
Education level	
Undergraduate/Graduate—Postgraduate	4.82 (1.11–21.00)
How interested are you in being able to share your experiences related to allergic rhinitis and communicate with other patients with the same condition through ICTs? (*n* = 217)	
Mean RQLQ score	1.40 (1.14–1.72)
Allergic rhinitis type^c^	
Mild persistent	12.59 (1.32–120.35)

*Note*: Binomial and ordinal regression analyses were adjusted for variables such as age, gender, education level, living area, allergic rhinitis type, years with allergic rhinitis, additional allergic condition and RQLQ score. All values are significant at 0.05 significance level.

Abbreviations: CI, confidence interval; OR, odds ratio.

^a^ Reference additional allergic condition category is “*No*”.

^b^ Reference education level category was “*Primary education*”.

^c^ Reference allergic rhinitis type was “*Mild intermittent*”.

Regarding the interaction with physicians, an increase in the mean RQLQ score was associated with an increase in the odds of being very interested in the use of ICTs for asking physicians questions about AR (OR, 1.27). A similar trend was also seen in patients with the highest educational category relative to those with the most basic education (OR, 5.29). Furthermore, patients with secondary education (OR, 0.13) and with undergraduate/graduate‐postgraduate studies (OR, 0.10) were less likely to consider that ICTs could reduce the need to see a physician.

An increase in the mean RQLQ score represented an increase in the interest of the development of AR content (OR, 1.23) and of apps to monitor symptoms and drug prescription (OR, 1.23). Also, the odds of individuals with the highest education level considering the app development for health‐related purposes was 4.82 times more than that of patients with the most basic education level. Interestingly, an increase in age was associated with a slight decreased chance (OR, 0.97) of being very interested in the development of these technologies. Finally, having a mild persistent disease (OR, 12.6) and/or a higher mean RQLQ score (OR, 1.40) were found to significantly predict greater likelihood of sharing experiences through ICT with other AR patients (Table 2).

## 4 DISCUSSION

This study showed that older patients found AR information from ICTs valuable. However, long‐term AR or coexistence of other allergic diseases decreased the probability of finding these techniques beneficial. Allergic Rhinitis patients with the lowest quality of life were 27% more likely to use ICTs to contact their doctor about their pathology and 23% more interested in AR‐related information. A higher academic level and worse quality of life may imply up to fourfold more interest in building an AR management and monitoring app. Academic preparation five‐fold enhanced the chance of utilizing ICTs to connect with their health provider but did not reduce doctor visits. Lower quality of life and mild‐persistent AR increased the likelihood of utilizing ICTs to exchange experiences and communicate with other patients by 12 times.

### 4.1 Top‐line results

In line with previous research, women were found to be the primary users of ICT for health‐related information.^3^, ^11^ The majority of participants in this study had moderate to severe AR, with intermittent and persistent types being the most common, greatly affecting their quality of life. This aligns with a study that revealed that about 50% of mHealth users with troublesome rhinitis or ocular symptoms reported work and quality of life impairment.^12^

### 4.2 The usefulness of ICT in obtaining information related to allergic rhinitis

The use of information and communication technologies (ICTs) for health‐related purposes has become increasingly popular in recent years. However, our study has found that there are significant variations in the use of ICTs based on age, education level, and the presence of an allergy‐related disease. Younger patients with higher academic levels and a greater impact of allergy‐related diseases on their quality of life tend to prefer using ICTs to interact with doctors. On the other hand, older patients are less likely to use ICTs for health‐related purposes. However, recent surveys have found that with a brief training session, the elderly's use of ICTs can be influenced positively.^13^

### 4.3 ICT usage differs depending on age, level of education, and quality of life

Several factors promote the use of ICTs for health‐related purposes, including younger age, higher education, living in urban areas, working, and continuing education. Additionally, those with chronic conditions in the family and a positive attitude toward teleconsultations, telediagnosis, and self‐medical records are more likely to use ICTs for health‐related purposes.^14^, ^15^ While Internet usage is most common among those aged 25–34,^16^ over half of the adolescents use the Internet to search for health‐related information.^17^ Additionally, education level influences the frequency of Internet usage for health‐related purposes, with individuals with higher education levels more likely to use ICTs for health‐related purposes.^18^ However, retired individuals and those with disabilities tend to use ICTs less frequently than professionally active individuals.^18^ Moreover, our study showed that the presence of allergy‐related diseases may decrease the likelihood of using AR‐related ICTs, even when controlling for age as a confounding factor. Understanding these factors can help healthcare providers better tailor their services to meet the needs of their patients and improve their outcomes.

### 4.4 Interest in participating in support groups and reaching other patients with AR

The use of ICTs has become an important tool for connecting people who have health‐related issues. Through online communities, patient‐centered websites, blogs, and social networks, patients can receive social support, empowerment, and interactive information‐emotion sharing that can positively impact their health behaviors and increase adherence to disease‐specific interventions.^7^ Participation in online forums is a popular activity among active Internet users, particularly for those with a higher RQLQ score or mild‐persistent disease in our reports. Studies have shown that the use of ICT support groups can improve the quality of life, mental health, active coping, and optimism.^19^ Many experiences of support through the use of ICTs have been described in various chronic diseases, including cancer, HIV/AIDS, depression, Alzheimer's, and debilitating illnesses. Online support communities offer patients the opportunity to share experiences with peers, reflect on living with a specific health condition, and become better informed about their health conditions while reducing feelings of loneliness and isolation.^20^, ^21^, ^22^, ^23^ The Health on Net Foundation Survey found that 60% of participants used online communities to be part of support groups.^24^

### 4.5 Strengths and limitations

Although the questionnaire used in this study was extensive, the sample size was relatively small. However, the study can still be useful in simplifying the questionnaire for a larger global study. The cross‐sectional nature of the study meant that cause‐and‐effect relationships could not be deduced. Additionally, the participants knew the purpose of the study, which may have introduced some responder bias. Even though the non‐probabilistic sampling could influence the external validity of our results, the inclusion criteria ensured that our participants shared certain characteristics, such as a confirmed diagnosis of AR and access to ICT and the Internet. This homogeneity can be advantageous in drawing specific conclusions related to this particular subgroup of patients. Nevertheless, this is the first study to assess preferences, perceptions, and attitudes related to ICTs among individuals with AR before the COVID‐19 pandemic. As telemedicine rapidly expands, our results may change. Finally, the study employed the validated RQLQ, which is commonly used by investigators and clinicians to assess the impact of interventions on real‐life situations and interpret meaningful clinical data.^25^

## 5 CONCLUSIONS

In conclusion, our study revealed that despite the potential benefits of ICTs in AR, Ecuadorian participants showed low AR‐related ICTs preferences. However, individuals with higher RQLQ scores, better education, and younger age were more likely to find ICTs beneficial, ask physicians about ICTs, prefer AR contents and apps through ICTs, and share AR experiences with other patients through support‐related ICT tools. Our findings suggest a digital gap between young and old as well as urban and rural locations where ICTs and e‐Health may have development potential. As such, allergists should use evidence‐based resources and establish new technology options for content, follow‐up, and emotional support for AR patients in Latin America.

## AUTHOR CONTRIBUTIONS

**Ivan Cherrez‐Ojeda**: Conceptualization (lead); data curation (lead); investigation (lead); methodology (lead); resources (lead); software (lead); supervision (lead); visualization (lead); writing—original draft (lead); writing—review and editing (lead). **Jean Bousquet**: Conceptualization (lead); investigation (lead); project administration (lead); resources (lead); supervision (lead); validation (lead); visualization (lead); writing—original draft (lead); writing—review and editing (lead). **Zouina Sarfraz**: Conceptualization (equal); investigation (equal); methodology (equal); resources (equal); writing—original draft (equal); writing—review and editing (equal). **Azza Sarfraz**: Conceptualization (equal); data curation (equal); investigation (equal); methodology (equal); resources (equal); supervision (equal); validation (equal); writing—original draft (equal); writing—review and editing (equal). **Monica Rodriguez Gonzales**: Conceptualization (equal); data curation (equal); methodology (equal); resources (equal); supervision (equal); validation (equal); writing—original draft (equal); writing—review and editing (equal). **Anna Bedbrook**: Conceptualization (equal); investigation (equal); methodology (equal); resources (equal); supervision (equal); validation (equal); writing—original draft (equal); writing—review and editing (equal). **Nelson Rosario**: Conceptualization (equal); investigation (equal); methodology (equal); resources (equal); validation (equal); visualization (equal); writing—original draft (equal); writing—review and editing (equal). **Benjamin Zepeda‐Ortega**: Conceptualization (equal); investigation (equal); methodology (equal); supervision (equal); writing—original draft (equal); writing—review and editing (equal). **Guillermo Guidos**: Conceptualization (equal); supervision (equal); validation (equal); visualization (equal); writing—original draft (equal); writing—review and editing (equal). **Ulbio Alcivar Molina**: Conceptualization (equal); investigation (equal); methodology (equal); project administration (equal); resources (equal); supervision (equal); validation (equal); visualization (equal); writing—original draft (equal); writing—review and editing (equal). **Miguel Felix**: Conceptualization (equal); data curation (equal); formal analysis (equal); investigation (equal); methodology (equal); project administration (equal); resources (equal); supervision (equal); validation (equal); writing—original draft (equal); writing—review and editing (equal). **Emanuel Vanegas**: Conceptualization (equal); data curation (lead); formal analysis (lead); investigation (equal); methodology (lead); project administration (equal); resources (equal); validation (lead); visualization (equal); writing—original draft (equal); writing—review and editing (equal). **Karla Robles‐Velasco**: Conceptualization (equal); investigation (equal); methodology (equal); resources (equal); supervision (equal); writing—original draft (lead); writing—review and editing (lead). **Luc J. Zimmermann**: Conceptualization (equal); investigation (equal); methodology (equal); project administration (equal); supervision (equal); writing—original draft (equal); writing—review and editing (equal). **Antonio W. D. Gavilanes**: Conceptualization (equal); methodology (equal); supervision (equal); validation (equal); visualization (equal); writing—original draft (equal); writing—review and editing (equal).

## CONFLICT OF INTEREST STATEMENT

The authors have no competing interests to disclose.

## Supporting information

Supporting Information S1

## Data Availability

The datasets used and/or analyzed during the current study are available from the corresponding author upon reasonable request.
