# Supplementary material for: Exploring the role of information and communication technologies in allergic rhinitis in specialist centers: Patient perspectives on usefulness, value, and impact on healthcare
Source: Clin Transl Allergy. 2024 Jan 19;14(1):e12325. doi: 10.1002/clt2.12325 (PMC10799206; doi:10.1002/clt2.12325)
Supplement: Supplementary file 1 — Supporting Information S1 [file CLT2-14-e12325-s001.docx]

| **Table S1 - Adjusted logistic regressions predicting the agreement/interest on ICT use for health-related purposes in allergic rhinitis according to age, years with allergic rhinitis and mean RQLQ.** | | | | | | | | | | | | | |
| --- | --- | --- | --- | --- | --- | --- | --- | --- | --- | --- | --- | --- | --- |
|  | **Age** | | | | **Years with allergic rhinitis diagnosis** | | | | **Mean RQLQ score** | | | | |
|  | Sig. | OR | 95% CI | | Sig. | OR | 95% CI | | Sig. | OR | 95% CI | |  |
|  |  |  | Lower | Upper |  |  | Lower | Upper |  |  | Lower | Upper |  |
| Have you used ICTs to obtain information about allergic rhinitis?^a^ (n=217) | .202 | 0.99 | 0.96 | 1.01 | .239 | 1.02 | 0.99 | 1.06 | .502 | 1.08 | 0.87 | 1.34 |  |
| Do you think that allergic rhinitis information obtained through ICTs is useful?^a^ (n=102) | .008 | 1.16 | 1.04 | 1.28 | .048 | 0.92 | 0.84 | 0.99 | .303 | 0.79 | 0.50 | 1.24 |  |
| How interested are you in asking questions to physician/medical health providers about allergic rhinitis through ICTs?^b^ (n=217) | .871 | 1.00 | 0.98 | 1.02 | .815 | 1.00 | 0.96 | 1.03 | .022 | 1.27 | 1.04 | 1.55 |  |
| Do you consider that ICTs could reduce the need to see a doctor?^a^ (n=217) | .324 | 0.99 | 0.96 | 1.01 | .156 | 1.03 | 0.99 | 1.07 | .786 | 0.97 | 0.75 | 1.24 |  |
| How interested are you in the development of content (articles, videos, tutorials, etc.) related to allergic rhinitis and allergies y general, created by general practitioners and specialists, accessible through ICTs?^b^ (n=217) | .774 | 1.00 | 0.98 | 1.03 | .405 | 1.02 | 0.98 | 1.05 | .049 | 1.23 | 1.00 | 1.52 |  |
| How interested are you in the development of an app designed to manage allergic rhinitis which allows to record your symptoms daily, including drug prescription reminders and general advice about the disease?^b^ (n=217) | .011 | 0.97 | 0.95 | 0.99 | .463 | 1.01 | 0.98 | 1.05 | .043 | 1.23 | 1.01 | 1.50 |  |
| How interested are you in being able to share your experiences related to allergic rhinitis and communicate with other patients with the same condition through ICTs?^b^ (n=217) | .063 | 1.02 | 1.00 | 1.04 | .573 | 0.99 | 0.96 | 1.02 | .001 | 1.40 | 1.14 | 1.72 |  |
| 1. Binomial logistic regressions adjusted for age, gender, education level, living area, allergic rhinitis type, years with allergic rhinitis, additional allergic conditions and RQLQ score. Reference category of dependent variable was "*No*". 2. Ordinal logistic regressions adjusted for age, gender, education level, living area, allergic rhinitis type, years with allergic rhinitis, additional allergic conditions and RQLQ score | | | | | | | | | | | | | |

**Supplemental Appendix**

| **Table S2 - Adjusted logistic regressions predicting the agreement/interest on ICT use for health-related purposes in allergic rhinitis according to gender.** | | | | |
| --- | --- | --- | --- | --- |
|  | Male | | | |
|  | Sig. | OR | 95% CI | |
|  |  |  | Lower | Upper |
| Have you used ICTs to obtain information about allergic rhinitis?^a^ (n=217) | .092 | 0.56 | 0.28 | 1.10 |
| Do you think that allergic rhinitis information obtained through ICTs is useful?^a^ (n=102) | .293 | 0.46 | 0.11 | 1.96 |
| How interested are you in asking questions to physician/medical health providers about allergic rhinitis through ICTs?^b^ (n=217) | .776 | 0.91 | 0.49 | 1.70 |
| Do you consider that ICTs could reduce the need to see a doctor?^a^ (n=217) | .491 | 0.75 | 0.34 | 1.69 |
| How interested are you in the development of content (articles, videos, tutorials, etc.) related to allergic rhinitis and allergies y general, created by general practitioners and specialists, accessible through ICTs?^b^ (n=217) | .794 | 0.92 | 0.48 | 1.75 |
| How interested are you in the development of an app designed to manage allergic rhinitis which allows to record your symptoms daily, including drug prescription reminders and general advice about the disease?^b^ (n=217) | .943 | 0.98 | 0.53 | 1.81 |
| How interested are you in being able to share your experiences related to allergic rhinitis and communicate with other patients with the same condition through ICTs?^b^ (n=217) | .838 | 0.94 | 0.51 | 1.74 |
| Notes: Reference category for gender was "*female*". ^a.^ Binomial logistic regressions adjusted for age, gender, education level, living area, allergic rhinitis type, years with allergic rhinitis, additional allergic conditions and RQLQ score. Reference category of dependent variable was "*No*". ^b.^ Ordinal logistic regressions adjusted for age, gender, education level, living area, allergic rhinitis type, years with allergic rhinitis, additional allergic conditions and RQLQ score. | | | | |

| **Table S3 - Adjusted logistic regressions predicting the agreement/interest on ICT use for health-related purposes in allergic rhinitis according to education level.** | | | | | | | | |
| --- | --- | --- | --- | --- | --- | --- | --- | --- |
|  | Secondary education | | | | Undergraduate/Graduate - Postgraduate | | | |
|  | Sig. | OR | 95% CI | | Sig. | OR | 95% CI | |
|  |  |  | Lower | Upper |  |  | Lower | Upper |
| Have you used ICTs to obtain information about allergic rhinitis?^a^ (n=217) | .482 | 1.89 | 0.32 | 11.17 | .197 | 3.19 | 0.55 | 18.61 |
| Do you think that allergic rhinitis information obtained through ICTs is useful?^a^ (n=102) | .999 | 0.00 | 0.00 | . | .999 | 0.00 | 0.00 | . |
| How interested are you in asking questions to physician/medical health providers about allergic rhinitis through ICTs?^b^ (n=217) | .310 | 2.18 | 0.48 | 9.82 | .030 | 5.29 | 1.18 | 23.72 |
| Do you consider that ICTs could reduce the need to see a doctor?^a^ (n=217) | .032 | 0.13 | 0.02 | 0.84 | .013 | 0.10 | 0.02 | 0.61 |
| How interested are you in the development of content (articles, videos, tutorials, etc.) related to allergic rhinitis and allergies y general, created by general practitioners and specialists, accessible through ICTs?^b^ (n=217) | .193 | 2.77 | 0.60 | 12.85 | .070 | 4.10 | 0.89 | 18.82 |
| How interested are you in the development of an app designed to manage allergic rhinitis which allows to record your symptoms daily, including drug prescription reminders and general advice about the disease?^b^ (n=217) | .128 | 3.16 | 0.72 | 13.92 | .036 | 4.82 | 1.11 | 21.00 |
| How interested are you in being able to share your experiences related to allergic rhinitis and communicate with other patients with the same condition through ICTs?^b^ (n=217) | .050 | 4.46 | 1.00 | 19.91 | .066 | 4.00 | 0.92 | 17.51 |
| Notes: Reference category for education level was "*primary education*". ^a.^ Binomial logistic regressions adjusted for age, gender, education level, living area, allergic rhinitis type, years with allergic rhinitis, additional allergic conditions and RQLQ score. Reference category of dependent variable was "*No*". ^b.^ Ordinal logistic regressions adjusted for age, gender, education level, living area, allergic rhinitis type, years with allergic rhinitis, additional allergic conditions and RQLQ score. | | | | | | | | |

| **Table S4 - Adjusted logistic regressions predicting the agreement/interest on ICT use for health-related purposes in allergic rhinitis according to living area.** | | | | |
| --- | --- | --- | --- | --- |
|  | Rural | | | |
|  | Sig. | OR | 95% CI | |
|  |  |  | Lower | Upper |
| Have you used ICTs to obtain information about allergic rhinitis?^a^ (n=217) | .380 | 0.45 | 0.08 | 2.68 |
| Do you think that allergic rhinitis information obtained through ICTs is useful?^a^ (n=102) | .577 | 0.40 | 0.02 | 9.95 |
| How interested are you in asking questions to physician/medical health providers about allergic rhinitis through ICTs?^b^ (n=217) | .697 | 0.75 | 0.17 | 3.23 |
| Do you consider that ICTs could reduce the need to see a doctor?^a^ (n=217) | .986 | 1.02 | 0.16 | 6.48 |
| How interested are you in the development of content (articles, videos, tutorials, etc.) related to allergic rhinitis and allergies y general, created by general practitioners and specialists, accessible through ICTs?^b^ (n=217) | .727 | 1.32 | 0.28 | 6.11 |
| How interested are you in the development of an app designed to manage allergic rhinitis which allows to record your symptoms daily, including drug prescription reminders and general advice about the disease?^b^ (n=217) | .422 | 1.84 | 0.42 | 8.15 |
| How interested are you in being able to share your experiences related to allergic rhinitis and communicate with other patients with the same condition through ICTs?^b^ (n=217) | .993 | 0.99 | 0.23 | 4.26 |
| Notes: Reference category for living area was "*urban*". ^a.^ Binomial logistic regressions adjusted for age, gender, education level, living area, allergic rhinitis type, years with allergic rhinitis, additional allergic conditions and RQLQ score. Reference category of dependent variable was "*No*". ^b.^ Ordinal logistic regressions adjusted for age, gender, education level, living area, allergic rhinitis type, years with allergic rhinitis, additional allergic conditions and RQLQ score. | | | | |

| **Table S5 - Adjusted logistic regressions predicting the agreement/interest on ICT use for health-related purposes in allergic rhinitis according to allergic rhinitis type (severity & pattern)** | | | | | | | | | | | | | |
| --- | --- | --- | --- | --- | --- | --- | --- | --- | --- | --- | --- | --- | --- |
|  | Mild persistent | | | | Severe intermittent | | | | Severe persistent | | | | |
|  | Sig. | OR | 95% CI | | Sig. | OR | 95% CI | | Sig. | OR | 95% CI | |  |
|  |  |  | Lower | Upper |  |  | Lower | Upper |  |  | Lower | Upper |  |
| Have you used ICTs to obtain information about allergic rhinitis?^a^ (n=217) | .594 | 1.92 | 0.18 | 20.87 | .914 | 0.94 | 0.28 | 3.11 | .881 | 0.91 | 0.27 | 3.13 |  |
| Do you think that allergic rhinitis information obtained through ICTs is useful?^a^ (n=102) | .999 | 242668119.50 | 0.00 | . | .801 | 1.38 | 0.11 | 17.10 | .334 | 0.30 | 0.03 | 3.44 |  |
| How interested are you in asking questions to physician/medical health providers about allergic rhinitis through ICTs?^b^ (n=217) | .080 | 7.30 | 0.79 | 67.40 | .664 | 1.27 | 0.43 | 3.75 | .928 | 1.05 | 0.35 | 3.19 |  |
| Do you consider that ICTs could reduce the need to see a doctor?^a^ (n=217) | .999 | 0.00 | 0.00 | . | .613 | 0.71 | 0.19 | 2.63 | .803 | 0.84 | 0.22 | 3.20 |  |
| How interested are you in the development of content (articles, videos, tutorials, etc.) related to allergic rhinitis and allergies y general, created by general practitioners and specialists, accessible through ICTs?^b^ (n=217) | .224 | 4.02 | 0.43 | 37.95 | .150 | 2.26 | 0.75 | 6.85 | .547 | 1.42 | 0.46 | 4.38 |  |
| How interested are you in the development of an app designed to manage allergic rhinitis which allows to record your symptoms daily, including drug prescription reminders and general advice about the disease?^b^ (n=217) | .765 | 0.72 | 0.09 | 5.97 | .437 | 1.53 | 0.52 | 4.50 | .613 | 1.33 | 0.44 | 4.00 |  |
| How interested are you in being able to share your experiences related to allergic rhinitis and communicate with other patients with the same condition through ICTs?^b^ (n=217) | .028 | 12.59 | 1.32 | 120.35 | .798 | 1.15 | 0.40 | 3.34 | .714 | 1.23 | 0.41 | 3.68 |  |
| Notes: Reference category for allergic rhinitis type was "*mild intermittent*". ^a.^ Binomial logistic regressions adjusted for age, gender, education level, living area, allergic rhinitis type, years with allergic rhinitis, additional allergic conditions and RQLQ score. Reference category of dependent variable was "*No*". ^b.^ Ordinal logistic regressions adjusted for age, gender, education level, living area, allergic rhinitis type, years with allergic rhinitis, additional allergic conditions and RQLQ score. | | | | | | | | | | | | | |

| **Table S6 - Adjusted logistic regressions predicting the agreement/interest on ICT use for health-related purposes in allergic rhinitis according to additional allergic condition.** | | | | |
| --- | --- | --- | --- | --- |
|  | Yes | | | |
|  | Sig. | OR | 95% CI | |
|  |  |  | Lower | Upper |
| Have you used ICTs to obtain information about allergic rhinitis?^a^ (n=217) | .007 | 0.40 | 0.21 | 0.78 |
| Do you think that allergic rhinitis information obtained through ICTs is useful?^a^ (n=102) | .041 | 11.86 | 1.11 | 127.06 |
| How interested are you in asking questions to physician/medical health providers about allergic rhinitis through ICTs?^b^ (n=217) | .837 | 1.06 | 0.59 | 1.91 |
| Do you consider that ICTs could reduce the need to see a doctor?^a^ (n=217) | .193 | 1.59 | 0.79 | 3.21 |
| How interested are you in the development of content (articles, videos, tutorials, etc.) related to allergic rhinitis and allergies y general, created by general practitioners and specialists, accessible through ICTs?^b^ (n=217) | .458 | 1.26 | 0.68 | 2.32 |
| How interested are you in the development of an app designed to manage allergic rhinitis which allows to record your symptoms daily, including drug prescription reminders and general advice about the disease?^b^ (n=217) | .452 | 1.25 | 0.70 | 2.24 |
| How interested are you in being able to share your experiences related to allergic rhinitis and communicate with other patients with the same condition through ICTs?^b^ (n=217) | .630 | 1.16 | 0.64 | 2.09 |
| Notes: Reference category for additional allergic condition was "*Yes*". ^a.^ Binomial logistic regressions adjusted for age, gender, education level, living area, allergic rhinitis type, years with allergic rhinitis, additional allergic conditions and RQLQ score. Reference category of dependent variable was "*No*". ^b.^ Ordinal logistic regressions adjusted for age, gender, education level, living area, allergic rhinitis type, years with allergic rhinitis, additional allergic conditions and RQLQ score. | | | | |
